# Endovascular Therapy for Intracranial Giant Cell Arteritis: Systematic Review, Technical Considerations and the Effect of Intra-arterial Calcium Channel Blockers

**DOI:** 10.1007/s00062-022-01171-0

**Published:** 2022-05-03

**Authors:** M. Travis Caton, Ian T. Mark, Kazim H. Narsinh, Amanda Baker, Daniel L. Cooke, Steven W. Hetts, Christopher F. Dowd, Van V. Halbach, Randall T. Higashida, Nerissa U. Ko, Sharon A. Chung, Matthew R. Amans

**Affiliations:** 1 Department of Radiology and Biomedical Imaging, Interventional Neuroradiology Section, University of California San Francisco, 505 Parnassus Ave, Room L349, 94143 San Francisco, CA USA; 2 Department of Neurology, University of California, San Francisco, USA; 3 Department of Medicine, Division of Rheumatology, University of California, San Francisco, USA

**Keywords:** Vasculitis, Cerebral ischemia, Angioplasty, Verapamil, Intracranial stenosis

## Abstract

**Background:**

Giant cell arteritis (GCA) is a systemic vasculitis that may cause ischemic stroke. Rarely, GCA can present with aggressive intracranial stenoses, which are refractory to medical therapy. Endovascular treatment (EVT) is a possible rescue strategy to prevent ischemic complications in intracranial GCA but the safety and efficacy of EVT in this setting are not well-described.

**Methods:**

A systematic literature review was performed to identify case reports and series with individual patient-level data describing EVT for intracranial GCA. The clinical course, therapeutic considerations, and technique of seven endovascular treatments in a single patient from the authors’ experience are presented.

**Results:**

The literature review identified 9 reports of 19 treatments, including percutaneous transluminal angioplasty (PTA) with or without stenting, in 14 patients (mean age 69.6 ± 6.3 years). Out of 12 patients 8 (66.7%) with sufficient data had > 1 pre-existing cardiovascular risk factor. All patients had infarction on MRI while on glucocorticoids and 7/14 (50%) progressed despite adjuvant immunosuppressive agents. Treatment was PTA alone in 15/19 (78.9%) cases and PTA + stent in 4/19 (21.1%). Repeat treatments were performed in 4/14 (28.6%) of patients (PTA-only). Non-flow limiting dissection was reported in 2/19 (10.5%) of treatments.

The indications, technical details, and results of PTA are discussed in a single illustrative case. We report the novel use of intra-arterial calcium channel blocker infusion (verapamil) as adjuvant to PTA and as monotherapy, resulting in immediate improvement in cerebral blood flow.

**Conclusion:**

Endovascular treatment, including PTA with or without stenting or calcium channel blocker infusion, may be effective therapies in medically refractory GCA with intracranial stenosis.

**Supplementary Information:**

The online version of this article (10.1007/s00062-022-01171-0) contains supplementary material, which is available to authorized users.

## Background

Giant cell arteritis (GCA) is an idiopathic, large vessel vasculitis which may cause flow-limiting stenosis of the intracranial arteries. GCA affects patients older than 50 years of age, with an estimated incidence of 10–30/100,000 [1]. Beyond the classical presenting symptoms of scalp tenderness, headache, and jaw claudication, the most feared cranial manifestation of GCA is visual loss seen in in 8–34% [2]. Ischemic stroke is a well-described but relatively rare complication in GCA, occurring in an estimated 2–7% [3, 4]. The majority of these ischemic complications are attributable to extracranial arterial disease resulting in thromboembolism or watershed ischemia [5]. By contrast, intracranial vessel luminal narrowing is rare [6]; however, for the subset of patients who develop severe intracranial manifestations of GCA, mortality despite maximum medical therapy may be as high as 58% [7].

**Fig. 1:**
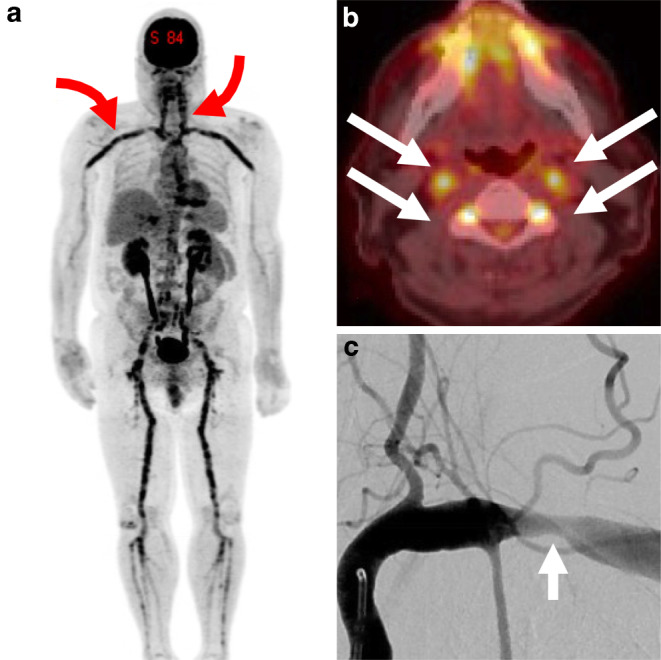
Manifestations of giant cell arteritis (GCA) with large-vessel involvement. FDG-PET maximum intensity projection reconstruction (**a**) shows florid hypermetabolism throughout the cervical and subclavian axillary arteries (*red arrows*) as well as the aorta, iliac, and femoral vessels. Axial fusion PET-CT image of the same patient (**b**) shows disease activity in both carotid and vertebral arteries (*white arrows*). Anteroposterior angiography of the left subclavian artery (**c**) shows classical appearance of extracranial circumferential large artery stenosis (*white arrow*)

Histopathologically, GCA manifests with inflammatory changes in all three arterial wall layers with granulomatous inflammation and “giant cells” at the intima-media junction, resulting in circumferential stenosis and hypermetabolism on 18F-FDG PET (Fig. 1a, b). Mononuclear cell migration is followed by destruction of elastic lamina and proliferation of medial smooth muscle cells (SMC) resulting in intimal hyperplasia and narrowing of the vessel lumen (Fig. 1c), accounting for ischemic symptoms throughout the body. In later phases, the inflammatory infiltrate dissipates, and SMC proliferation is replaced with fibrosis. Large vessel involvement was once thought to be uncommon in GCA but is now recognized in 2/3 of patients including the involvement of the brachiocephalic (47.5%), cervical carotid (35%), and subclavian arteries (42.5%) [8]. More recent work using high-sensitivity MR vessel-wall imaging also found higher than expected rates of intracranial arterial involvement (up to 40%) [9].

Medical therapy with aggressive glucocorticoids and adjuvant therapy with other immunosuppressive agents remains standard of care in GCA [2, 10]. Aspirin has been shown to reduce intracranial ischemic events in GCA via both platelet inhibition and a proposed secondary mechanism of suppression of interferon gamma signaling, but its efficacy has not been proven in randomized trials [11, 12]. Despite the overall efficacy of medical therapy, a subset of patients develop refractory neurologic deficits and cerebral infarcts.

Percutaneous transluminal angioplasty (PTA) was first reported as a treatment for GCA involving the axillary arteries. Although data are limited, PTA appears safe and efficacious in peripheral vessels but its durability is uncertain [13]; restenosis is estimated to occur in 33% after single session treatment and 18.4% after repeat treatment [14]. Reports of post-PTA stent placement in large vessel vasculitides are even more sparse [15]. The stenoses of intracranial GCA are appealing targets for endovascular treatment; however, the risk of spontaneous dissection in this population is considerable with an estimated incidence of 25% [16]. Given the uncertain efficacy and high potential risk, endovascular treatment of intracranial GCA is rarely described in the medical literature.

The purpose of this study was to investigate the indications, safety profile, and clinical efficacy of endovascular treatment for medically refractory intracranial GCA. To this end, the authors present the technical experience of a single case of severe and refractory intracranial GCA which required seven endovascular treatments that included intra-arterial calcium channel blocker infusion, which has not previously been reported in this setting. Lastly, the authors present a systematic review of the existing literature on endovascular management of GCA.

## Material and Methods

The authors performed a systematic review of EMBASE, Medline, and Web of Science online databases through January 2021 in accordance with PRISMA guidelines [17]. Studies which reported any endovascular treatment (angioplasty, stent, intra-arterial drug infusion) to address symptoms attributed to confirmed GCA (diagnosed in accordance with EULAR-ACR criteria). Studies of non-GCA vasculitides were excluded. The literature search strategy is included as Supplemental Fig. 1. Duplicate references were removed, and references were manually reviewed to identify additional studies of interest.

The clinical and technical details of a single patient treated for medically refractory GCA treated at our institution were reviewed in detail and technical aspects about the endovascular treatment, including decision thresholds for treatment, are presented and discussed in depth. After each treatment with PTA, post-procedure blood pressure control was achieved with intravenous nicardipine infusion with target systolic pressure ≤ 120 mm Hg.

## Results

### Technical Case Report

**Fig. 2:**
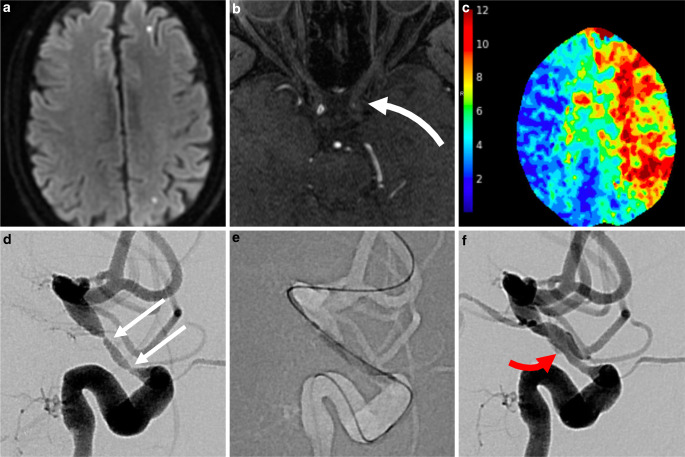
Intracranial manifestations of giant cell arteritis with left ACA-MCA watershed ischemia on DWI MRI (**a**). Time-of-flight MR angiography showed high-grade stenosis of supraclinoid LICA (*white curved arrow*, **b**). CT perfusion showed at-risk parenchyma throughout the left hemisphere, shown as T_max_ map (**c**) which prompted urgent endovascular treatment (treatment #1). Pretreatment lateral projection LICA injection shows 2 sites of critical stenosis (**d**, *white arrows*) which were treated with submaximal balloon angioplasty (**e**). Posttreatment result **f** showed substantial improvement in angiographic transit time and return of antegrade flow to the ophthalmic and posterior communicating arteries. Careful review of images showed a tiny, non-flow limiting dissection measuring < 1 cm (*red arrow*)

A patient presented with 1 month of bitemporal headaches, night sweats, chills, and weight loss. There were no visual symptoms. Erythrocyte sedimentation rate (ESR) was > 80 mm/h and C‑reactive protein (CRP) of > 35 mg/dl. A temporal artery biopsy showed a marked chronic inflammatory infiltrate in the media of the artery with multiple foci of fibrinoid necrosis and multiple granulomas in the media of the artery wall, confirming the diagnosis of GCA. This finding was corroborated with widespread large vessel inflammation on 18F-FDG PET (Fig. 1). Glucocorticoid therapy was initiated (60 mg prednisone with 10-day tapered dose). Magnetic resonance imaging (MRI) showed scant foci of ischemia in the left anterior and middle cerebral artery (ACA, MCA) superficial watershed (Fig. 2a). On day 13 of treatment, the patient developed right hand weakness and word-finding difficulties prompting hospital admission and pulse-dose glucocorticoids (1 g methylprednisolone daily for 3 days) followed by high-dose oral glucocorticoids. This was augmented with tocilizumab 2 mg/kg intravenously per week. Language deficits progressed on day 19 and a repeat MRI showed new white matter watershed infarct with magnetic resonance angiography (MRA) showing critical stenosis of left internal carotid artery (ICA) and left intradural vertebral artery (VA). Tocilizumab was discontinued and intravenous pulse cyclophosphamide using the CYCLOPs dosing regimen [18] was initiated along with atorvastatin (80 mg daily) and clopidogrel (75 mg daily).

On day 28, due to progressive aphasia and weakness, the patient was taken for endovascular treatment, the first of seven such treatments performed (detailed in Table 1). In each case, the decision to treat was driven by severe vessel stenosis (≥ 70%) and one or more of the following: new ischemic lesions on MRI (treatments 1, 2, 5, 7), prolonged CT perfusion T_max_ (treatments 3, 4), or abrupt decline in neurologic examination (treatments 1–5, 7). Sub-maximal PTA was performed using coaxial guide catheter support with either the Cook Shuttle 6 Fr Sheath or Benchmark 0.071″ guide catheter and the Gateway Balloon System (Stryker Neurovascular, Kalamazoo, MI, USA) with gradual inflation (~1 atm. per 15–30 s) to nominal pressure (6 atm.). An angiographic result of < 50% stenosis was considered technically successful. In each case, delayed (15 min) angiography was performed after PTA to confirm absence of dissection and acute restenosis. A small, non-flow limiting dissection occurred after the first PTA of LICA (Fig. 2f); based on this complication, the decision was made to defer post-PTA stent placement in subsequent treatment sessions as the additional risk conferred by stenting was felt to be prohibitive. Dual antiplatelet therapy (aspirin 81 mg and clopidogrel 75 mg) was initiated at this time with concurrent gastrointestinal mucosal protection (famotidine 20 mg, daily).

**Table 1:** Summary of Seven Endovascular Treatments Performed for Refractory Giant Cell Arteritis with Intracranial Stenosis

Endovascular treatments in medically refractory intracranial giant cell arteritis in a single patient							
Treatment number (date from start of medical therapy)							
	1 (Day 28)	2 (Day 38)	3 (Day 48)	4 (Day 49)	5 (Day 67)	6 (Day 80)	7 (Day 136)
Indication for treatment	LICA/ACA watershed stroke DWI	Pontine Stroke DWI, prolonged CTP Tmax	Pressure-dependent LLE weakness, prolonged CTP Tmax	RLE weakness	New left ACA/ICA watershed infarct DWI, BLE weakness	New cerebellar infarct DWI	New left basal ganglia infarct DWI, decreased right sided movement
Target vessel(s)	LICA (PTA + CCB)	LVA (PTA + CCB)	LICA (PTA + CCB), RICA (CCB)	RICA (PTA + CCB)	LICA, supraclinoid (PTA + CCB) LICA, petrous (PTA + CCB)	LVA (PTA + CCB)	LICA (PTA)
Devices	2.0 × 15 mm Gateway Balloon	2.0 × 15 mm Gateway Balloon	2.25 × 15 mm Gateway Balloon	2.5 × 15 mm Gateway Balloon	3.0 × 15 mm (petrous) and 2.5 × 9 mm Gateway Balloon (supraclinoid)	2.0 × 15 mm Gateway Balloon	3 × 20 mm and 2.25 × 20 mm Gateway Balloon
Verapamil	5 mg, LICA	10 mg, LVA	20 mg, RICA 15 mg, LICA	10 mg, RICA	10 mg, LICA	20 mg, LVA	None
Complications	Non-flow limiting dissection	–	–	–	AICH	–	AICH
Angiographic result	> 90% → 50% stenosis	LVA 90% → 50%	RICA: 80% → 60% LICA: > 90% → 60%	RICA 90% → 30%	LICA petrous > 90% → 20% LICA supraclinoid 90% → 40%	LVA 2 sites, both 80–90% → 20%	LICA > 90% → 20%
Treatment durability^a^	–	LICA: durable	LVA: durable LICA: restenosis RICA: progression	RICA: restenosis LICA: durable LVA: durable	Petrous LICA: new stenosis supraclinoid LICA: restenosis RICA: durable LVA: durable	LVA: restenosis RICA: durable LICA: durable	LICA restenosis LVA: durable RICA: durable

*AICH* asymptomatic intracranial hemorrhage, *LICA* left internal carotid artery, *RICA* right internal carotid artery, *ACA* anterior cerebral artery, *PTA* percutaneous transluminal angioplasty, *LVA* lift vertebral artery, *CCB* calcium channel blocker, *LLE* left leg, *RLE* right leg, *BLE* both leg, *DWI* diffusion weighted imaging

^a^Defined by luminal diameter on subsequent DSA compared with immediate prior DSA

**Fig. 3:**
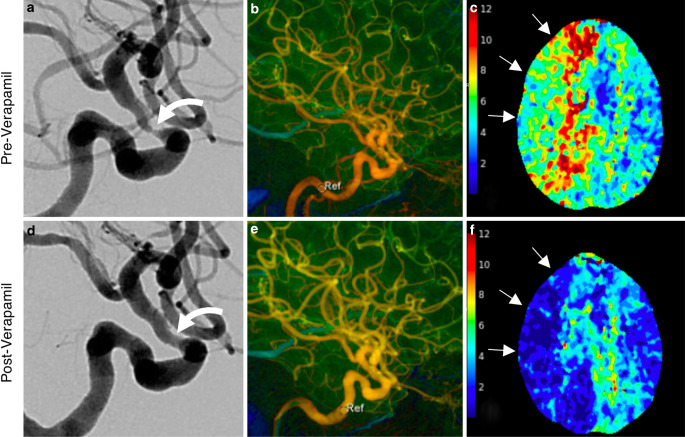
Calcium channel blocker infusion as monotherapy for intracranial giant cell arteritis. Pre-treatment angiography (lateral RICA projection) shows severe focal supraclinoid segment stenosis (**a**). Color-coded four-dimensional DSA (4D-DSA, **b**) shows prolonged transit time throughout the RICA circulation; sample velocity at the petrous segment time-to-peak (TTP) velocity of 4.53s. CTP performed the day prior to intervention showed at-risk tissue (prolonged T_max_) throughout the right ICA territory (**c**, *arrows*). Post-verapamil infusion (20 mg, 15 min delay) angiogram is shown in **d**, with significant improvement in lumen diameter. Post-verapamil 4D-DSA (**e**) shows improved flow throughout the ICA distribution and normalization of TTP in the petrous segment (1.0s) (*circle*, labeled *Ref*). A CTP performed 10 h after verapamil infusion shows durable improvement in T_max_ in RICA distribution (**f**)

Notably, during the third treatment (hospital day 48), flow-limiting stenosis (70%) of the right supraclinoid ICA was treated with intra-arterial (IA) verapamil infusion (20 mg given over 15 min) alone resulting in significant improvement in ICA caliber and flow (Fig. 3). The resultant improvement in transit time persisted on a CTA/CT perfusion study performed 10 h after the procedure. The patient was subsequently started on daily oral nimodipine.

Two days after the second treatment, left leg weakness developed and a CTA showed worsening right ACA hypoperfusion. Therefore, the patient was treated the following day (hospital day 49) with RICA PTA and IA verapamil (20 mg over 15 min) with good angiographic effect and no complications (treatment 4). The patient was discharged to rehabilitation 2 weeks later but was readmitted (day 66) with new watershed LICA stroke with restenosis of the supraclinoid segment (80%), which was treated (day 67) with PTA and 10 mg IA verapamil. Cyclophosphamide was discontinued and treatment with tocilizumab was initiated but ultimately discontinued due to small bowel and gall bladder pneumatosis, a well-known complication of tocilizumab [19]. On day 77, brain MRI showed new punctate cerebellar infarct and a CTA showed prolonged mean transit time in the vertebrobasilar territory. Subsequent DSA showed 2 foci of > 90% stenosis in the V4 segment, which were treated with PTA. During that procedure (treatment 6, day 80), the left and right ICA showed stable post-PTA caliber. Head CT 24 h later showed asymptomatic hemorrhage in the left thalamus. Over the following 7 weeks, treatment with prednisone continued with adjuvant treatment with intravenous abatacept (10 mg/kg), followed by a glucocorticoid taper. Dual antiplatelet therapy was discontinued on day 93 due to lower gastrointestinal bleeding. Surveillance MRI/MRA was performed on day 135 showing new left basal ganglia infarction despite a plateau in symptoms along with restenosis of supraclinoid LICA and new stenosis of petrous segment of LICA. PTA of both stenotic segments was performed (treatment 7) on the following day with marked caliber improvement; IA verapamil was deferred, and blood pressure goals were reduced to mitigate reperfusion injury. Despite these precautions, another asymptomatic intracranial hemorrhage (ICH) of the basal ganglia and remotely infarcted cingulate cortex was seen on next-day CT. The patient’s symptoms gradually improved but residual deficits attributable to ACA infarct and left thalamic injury were present on discharge.

### Literature Review

Initial query of the databases identified 55 studies after duplicates were removed. Of these, nine studies were included in the analysis [20–28] after excluding cases in which GCA was not the diagnosis or the endovascular treatment did not involve intracranial vessels. The demographic, clinical, and technical details of each study are shown in Table 2.

**Table 2:** Systematic Literature Review for Endovascular Treatment for Intracranial Giant Cell Arteritis

Article	Author	Patient Number	Sex	No. of Treatments	Technique	Target Vessel	CV Risk Factors	ESR	CRP	Corticosteroid treatment^a^	Adjuvant Immunosuppressant^a^	Vascular medications^a^	Complications	Clinical outcome^b^	Clinical follow-up (days)
1	Simonsen et al. 2020 [20]	1	65F	2	PTA	LICA	None	46	NR	Prednisone 1 mg/kg → solumedrol → prednisone taper	CPM 15 mg/kg → tocilizumab → oral CPM × 6 mo, then methotrexate	NR	1	MRS 2	90
		2	64F	3	PTA	LICAx3, RICAx2	HTN, DM	57	NR	Prednisone 1 mg/kg → solumedrol →	CPM 15 mg/kg × 6 → methotrexate	NR	Dissection x1, PCP pneumonia	MRS 3	90
		3	72M	2	PTA	LICAx3, RICAx1, LVx1	HTN, smoking	22	NR	Prednisone taper 1 mg/kg → solumedrol → prednisone taper	CPM 10 mg/kg × 6	NR	0	MRS 1	90
		4	71F	2	PTA	LICAx2, RICAx2, LVx1	HTN	54	NR	Solumedrol 1 mg/d →	CPM 15 mg/kg × 6	NR	Dissection x1	MRS 4	90
2	Lago et al. 2020 [21]	5	72F	1	PTA	LICA	HTN	50	NR	Prednisone, ‘megadose’steroids dose NR	None	ASA during hospitalization	0	MRS 2	90
		6	73M	1	PTA	Bilateral VA	HTN, DM, smoking	68	49.5	Prednisone, dose NR	None	None	0	MRS 1	90
3	Togo et al. 2018 [22]	7	80F	1	PTA	R VA	None	90	7.5	1 g solumedrol → prednisone → 1 g solumedrol	CPM 670 mg	ASA 100 mg/clopidogrel 75 mg on HD1	0	MRS 3	71
4	Guerrero et al. 2015 [23]	8	59F	1	PTA + Stent	LICA	None	101	NR	1 g solumedrol × 3 day → prednisone 1 mg/kg	Methotrexate 7.5 mg/weekly started week 2	ASA 100 mg on HD 0, then enoxoparin BID-DAPT post stent	0	MRS 2	32
5	Neutel et al. 2014 [24]	9	65M	1	PTA	LICA	HTN, HLD	57	NR	Prednisone (taper dose) → 1 mg/kg → bolus → taper	Methotrexate (15 mg/week)	ASA 100 on HD 0, switched to plavix 75 mg monotherapy after recurrent stroke	0	NR	365
6	Dementovych et al. 2012 [25]	10	NR	1	PTA + Stent	R VA	HTN, HLD, DM	51	28	Solumedrol 1 mg/d → prednisone	None	Clopidogrel 300/ASA 325 (day 21 stroke); DAPT post stent	0	NIHSS 3 (from 13) at day 30	30
7	Chausson et al. 2010 [26]	11	78F	1	PTA + Stent	Bilateral ICA	None	99	25	Solumedrol 1 mg/d → prednisone	None	Clopidogrel (day of stroke, HD31) → DAPT post stent	0	NR	7
8	Steiger et al. 2018 [27]	12	67M	1	PTA + Stent	R VA	HTN, HLD, DM, CAD	93	14	Solumedrol 1 mg/d → prednisone	None	ASA, then DAPT and AC (Not specified) day 16 stroke	0	MRS 1	60
9	Espígol-Frigolé et al. 2009 [28]	13	NR	1	PTA	NR (BICA/BVA)	Unknown	‘Elevated’	NR	Solumedrol, prednisone (dose NR)	None	Antiplatelet and anticoagulation (dose/type NR)	0	MRS 0	540
		14	NR	1	PTA	NR (BICA)	Unknown	NR	NR	Solumedrol, prednisone (dose NR)	None	Antiplatelet and anticoagulation (dose/type NR)	0	MRS 0	540

*NR* not reported, *PTA* percutaneous transluminal angioplasty, *ESR* erythrocyte sedimentation rate (s), *CRP* C-reactive protein (mg/dl), *ICA* intracranial internal carotid artery, *VA* vertebral artery, *HTN* hypertension, *DM* diabetes mellitus, *AC* anticoagulation, *ASA* aspirin, *DAPT* dual antiplatelet therapy, *MRS* modified Rankin Scale, *CPM* cyclophosphamide

^a^Indicates prior to endovascular intervention

^b^MRS as directly reported in study or inferred from clinical description

### Clinical, Demographic and Diagnostic Characteristics

In the selected studies, 14 patients underwent a total of 19 endovascular procedures. These comprised individual treatments of 32 vessels: 71.9% (23/32) of the internal carotid artery (ICA) and 28.1% (9/32) of the vertebral arteries. The diagnosis of GCA was confirmed by temporal artery biopsy in 12 of 14 patients and the remaining 2 patients were diagnosed by the American College of Rheumatology criteria [29]. Mean ESR at time of diagnosis was 65.7 ± 24.7 mm/h and mean CRP concentration was 19.5 ± 7.4 mg/dl. 18F-FDG PET imaging showed evidence of arterial wall hypermetabolism in 4/14 patients, was normal in 2/14 patients, and was not reported for the remaining patients. Brain MRI confirmed cerebral infarct on diffusion-weighted imaging (DWI) in all cases.

Demographic information was available for 11/14 patients. Mean patient age was 69.6 ± 6.3 years and 7/11 (63.4%) were women. Clinical risk factor data were available for 12/14 patients. The majority of patients (8/12, 66.7%) had at least one pre-existing cardiovascular risk factor: 8/12 (66.7%) had hypertension, 5/12 (41.2%) had type 2 diabetes mellitus, 3/12 (25%) had dyslipidemia, and 2/12 (16.7%) were current tobacco smokers.

### Medical Treatment

All patients were treated with glucocorticoid therapy prior to endovascular treatment, comprising high-dose prednisone with or without pulse dose of methylprednisolone. 50% (7/14) of patients were also started on an additional immunosuppressant due to refractory symptoms. Of these, 5/14 were treated with intravenous cyclophosphamide (10–15 mg/kg), 4/14 with methotrexate (7.5–15 mg/week), and 1/14 with tocilizumab.

Antiplatelet agents and/or anticoagulation were described for 10/14 cases. A single antiplatelet agent (either aspirin or clopidogrel) was reported as part of initial treatment in 5/14 patients. Three patients were managed with DAPT after a confirmed imaging diagnosis of stroke and prior to endovascular treatment. Four patients were treated with an anticoagulant (enoxaparin or heparin) after the diagnosis of stroke. All patients treated with stents were treated with DAPT following the procedure.

### Endovascular Treatment

None of the patients received intra-arterial verapamil or other vasodilators and78.9% (15/19) of endovascular treatments involved PTA alone. The remaining 21.0% (4/19) of treatments, representing 28.6% (4/14) of all patients, employed PTA followed by stent placement: 1 with 4 overlapping Wingspan stents (Stryker), 1 with 2 overlapping Enterprise Stents (Cordis Neurovascular, Miami, FL, USA), 1 with a Tecnic Carbostent (a balloon-mounted coronary stent, Sorin Biomedia, Saluggia, Italy). PTA was performed using a balloon in all but two cases, in which the Comaneci Device (Rapid Medical, Yokneam, Israel) was used. In total, 10/14 (71.4%) of patients underwent a single endovascular treatment and the remaining 4/14 (28.6%) had repeat treatments, including 1 patient treated 3 times.

Immediate angiographic improvement in luminal diameter was reported in all cases. Procedure-related arterial dissection was reported in 10.5% (2/19) of cases though none were associated with new neurologic deficits or reported long-term sequelae.

The overall mean follow-up duration was 157 days. Clinical outcomes were reported directly as modified Rankin score (MRS) at 90-day follow-up for 6 patients (Table 2). MRS was reported at < 90 intervals for 3 patients and was inferred for the remaining 4 patients based on provided clinical data. In 1 patient, NIH stroke scale improved to 3 from 13 at 30-day follow-up.

## Discussion

Intracranial arterial stenosis due to GCA is potentially a life-threatening condition. When available anti-inflammatory and immunosuppressive therapies are ineffective, endovascular treatment can prevent catastrophic cerebral infarction. Emerging evidence has resulted in the inclusion of endovascular therapy in the revised European League Against Rheumatism (EULAR) guidelines for the first time (a 4C recommendation), noting that unless emergent, such procedures should be deferred until disease activity is stabilized [30]. Accordingly, the authors believe that practicing neurointerventionalists should understand the risks, indications, and therapeutic considerations of treating intracranial GCA.

The paucity of data on endovascular therapy for intracranial GCA is a testament to the anticipated risk of intervention in this population. Our review identified reports of only 14 patients in the literature. By synthesizing these cases, we identified several important themes. First, endovascular treatment carries significant procedural risks but may benefit patients with refractory, flow-dependent neurologic symptoms, or recurrent stroke despite maximal medical therapy. In our experience, asymptomatic dissection occurred in 1/8 (12.5%) symptomatic stenosis treated with PTA compared with 10.5% from the literature review. Intraprocedural dissection is relatively common in PTA of intracranial arteries for atherosclerotic disease and despite slow balloon inflation and proper balloon sizing, it occurs in 14–18% of cases [31]. The use of stents following PTA likely conveys an additional risk to PTA alone, although the marginal risk is difficult to estimate [32]. Contemporary estimates of intracranial PTA with or without stenting for atherosclerotic lesions show substantial morbidity, with the non-stroke periprocedural complication rate ranging from 6.4–13.4% and the periprocedural stroke rate of 9.5% (7–12%) [33]. The risk of PTA and stenting in GCA is presumably higher due to vessel wall inflammation and predisposition to spontaneous dissection in extracranial vessels in these patients [16]. GCA stenoses are histologically associated with overexpression of platelet-derived growth factor, a key driver of intimal hyperplasia, which adds to the theoretical risk of delayed stenosis of intracranial stents in this population [34]. Despite these risks, we noted a substantial repeat treatment rate (28.5% of patients) with the PTA only strategy compared with 0% in stented patients. The risk of a repeat procedure must therefore be weighed against the risks of stent-related complications and DAPT.

We also encountered two (25.0%) delayed, postprocedural intraparenchymal hemorrhages in the same territory as PTA. In both instances, immediate postprocedure Dyna-CT showed no hemorrhage, but hemorrhage occurred within 24 h. The mechanism in both cases was therefore attributed to rapid reperfusion (within subacute infarcts in one case and de novo infarct in the other). Reperfusion hemorrhage is a well-described but infrequent complication of PTA in intracranial atherosclerotic disease (ICAD) and reported in 3.3% of cases from the SAMMPRIS trial. [35] Unlike ICAD which affects both large and small vessels, GCA is an isolated large vessel arteritis with presumably normal microvascular flow and intact autoregulatory capacity. Rapid correction of flow-limiting stenosis, particularly when stenoses are present in additional vascular territories, is a plausible mechanism that may account for the higher rate of postprocedural ICH in our case, as has been reported in cervical ICA intervention [36]. Prompt reduction of blood pressure after reperfusion may be appropriate for EVT in GCA, as this technique is associated with lower rates of spontaneous intracerebral hemorrhage (ICH) and better functional outcomes in acute stroke intervention [37]. The authors believe that periprocedural blood pressure control should be tailored on a case-by-case basis, generally targeting a systolic pressure ≤ 100 mm Hg. Novel post-PTA angiographic biomarkers such as early draining vein and capillary blush may also aid in blood pressure target selection [38]. Although not entirely clear from the present study, PTA without adjunctive stenting showed lower rates of postprocedural ICH in one prospective study [32]. Both of these strategies should be considered when endovascular treatment of GCA is undertaken.

This review also highlights that the majority of patients who required endovascular treatment (66.7%) had at least one pre-existing cardiovascular risk factor. This suggests that co-morbid GCA and cardiovascular disease synergistically increase stroke risk, and that such patients require optimization of statins, antihypertensives, and antiplatelet agents. The use of antiplatelet agents in GCA with either visual symptoms or stroke has a contentious history, as evident in the inconsistent use of antiplatelet agents in the current literature review. The 2009 European League Against Rheumatism (EULAR) guidelines recommended empiric low dose aspirin in patients diagnosed with GCA based on retrospective data from two large studies [10]. A 2014 meta-analysis on the effect of antiplatelet and anticoagulation on ischemic events in GCA found no protective benefit for patients prior to diagnosis but found a marginal event reduction when antiplatelet and/or anticoagulants were instituted after diagnosis (odds ratio, OR 0.318 (0101–0.996), *p* = 0.049) [39]. Consequently, the 2018 update to the EULAR guidelines reversed the original recommendation, citing the results of subsequent studies which showed no benefit to prophylactic antiplatelet therapy [30]. More recently, the 2021 American College of Rheumatology guidelines provided a conditional recommendation for aspirin only for patients with flow-limiting vertebral or carotid artery stenosis [40]. In our review, most patients were managed with a single antiplatelet agent although treatment was highly variable (Table 2). Aspirin, compared with other antiplatelet agents, offers the additive benefit of inhibiting interferon gamma signaling, an important mediator of disease severity in GCA and a pathway which is not directly inhibited by glucocorticoids alone [41]. Dual antiplatelet therapy (DAPT) has not been well-studied in this population. Currently, the 2022 American Academy of Neurology Clinical Practice Guidelines recommend DAPT as first-line therapy for symptomatic intracranial atherosclerosis to prevent ischemic stroke [42]; however, given the pathogenetic differences between radiographic stenoses in intracranial atherosclerosis and GCA, it is unclear if the protective effects of DAPT seen in intracranial atherosclerosis translate to intracranial GCA. Moreover, the risk of gastrointestinal hemorrhage (as in our case) or surgical complications related to bowel perforation in tocilizumab-treated patients are important factors when DAPT is considered [19].

In this review, patients with aggressive intracranial GCA typically did not respond to conventional glucocorticoid therapy and, 50% of cases progressed despite escalating therapy with immunomodulatory agents. This suggests that patients who develop refractory intracranial GCA differ from typical GCA patients but the reason for this is unclear. It is known that patients with more aggressive inflammatory responses (IL‑6, IL1-beta, and TNF-alpha) require longer duration of glucocorticoids to achieve remission [43]. It has also been observed that some elements of the inflammatory response, particularly neoangiogenesis, are associated with lower rates of ischemic complications and that patients who harbor VEGF mutations more frequently have severe ischemic complications [44]. Interestingly, patients who develop stroke typically do so following initiation of glucocorticoid therapy [3]. Together, these observations have led these investigators to hypothesize that inflammation-mediated neoangiogenesis is protective against ischemic events. Taken together, these data suggest that a select subset of GCA patients who show clinically severe disease with relatively milder than expected laboratory inflammatory markers may be particularly vulnerable to refractory large vessel stenoses and associated complications.

The use of IA calcium channel blocker (CCB) infusion is a novel treatment strategy for intracranial GCA. IA verapamil infusion is an established modality for vasospasm due to aneurysmal subarachnoid hemorrhage (aSAH) and serves as another potential rescue therapy in GCA when PTA is contraindicated or technically not feasible. The vasodilatory action of verapamil in aSAH vasospasm is likely multifactorial, but its principle mechanism is to induce vascular SMC relaxation via inhibition of L‑type calcium channel influx [45]. The pathogenesis of arterial luminal narrowing in GCA differs from SAH vasospasm but also appears to involve vascular SMC. A 4-phase model of vessel stenosis in GCA has been proposed, in which an inciting event stimulates activity in adventitial dendritic cells. Next, a cascade of inflammatory signals recruits and activates CD4+ cells and later, CD8+ cells and monocytes, the latter of which mature into macrophage-like multinucleated “giant” cells (MGCs) [46]. MGCs, via VEGF and PDGF signaling pathways [34], stimulate migration and proliferation of smooth muscle cells, resulting in vasoconstriction in the acute phase, followed by progressive intimal hyperplasia and eventually vaso-occlusive disease. Together, these observations implicate SMC relaxation as the common final pathway for the vasodilatory actions of CCB in both aSAH and the acute phase of GCA. The authors speculate that CCBs are unlikely to be effective in the later stages of GCA, which are characterized by fibroproliferative change. Nonetheless, drawing on decades of experience and level 1 evidence supporting the use of CCB in aSAH vasospasm, CCB may prove a valuable salvage therapy for patients with GCA and recurrent stroke, although future research is needed [47].

Several limitations of this review warrant additional discussion. First, the limited number of cases reported in the literature is insufficient to draw robust practice guidelines for neurointerventional care of GCA. Moreover, owing to publication bias, periprocedural complication rates may be underreported in this review. Heterogeneous reporting of inflammatory markers, clinical follow-up, and outcomes, as well as angiographic findings (i.e. degree of stenosis pretreatment and posttreatment) further limit this study. One major unanswered question is whether concomitant vessel stenting is advantageous or potentially harmful. The authors experience with variable durability of PTA and the need for multiple retreatments (restenosis rate of 77%) could be interpreted as evidence that the upfront risk of stenting may be favorable to the risks inherent to multiple retreatments. It should be noted that the present case differed from most in the literature review in that symptomatic stenosis and or infarct was seen in three large vessel territories, thus potential risk of collateral failure was considerable and influenced treatment decision-making in a manner different than cases with a single affected vascular territory. Finally, GCA stenoses are histologically associated with overexpression of platelet-derived growth factor, a key driver of intimal hyperplasia, which adds to the theoretical risk of stenting in this population [34].

In summary, endovascular management plays an important role in the management of severe and refractory intracranial GCA. PTA can be performed and repeated, if necessary, when stenting is deemed too risky. The risks of these mechanical interventions are considerable, and they should be used sparingly in patients with recurrent ischemic events, despite maximal medical therapy. Intra-arterial calcium channel blocker infusion offers a novel, less invasive treatment option for select patients.

## Supplementary Information

Supplemental Figure 1: PRISMA IPD Flow Diagram
