# Supplementary material for: Endovascular Therapy for Intracranial Giant Cell Arteritis: Systematic Review, Technical Considerations and the Effect of Intra-arterial Calcium Channel Blockers
Source: Clin Neuroradiol. 2022 May 3;32(4):1045–56. doi: 10.1007/s00062-022-01171-0 (PMC9744710; doi:10.1007/s00062-022-01171-0)
Supplement: Supplementary file 1 — Supplemental Figure 1: PRISMA IPD Flow Diagram [file 62_2022_1171_MOESM1_ESM.docx]

**Supplemental Figure 1:**


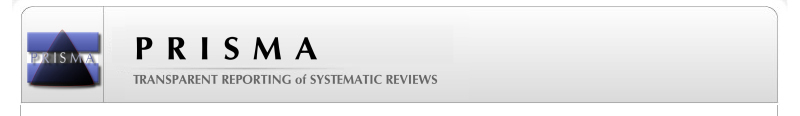


**PRISMA IPD Flow Diagram**

123 studies identified through database search (Web of Science, EMBASE, MEDLINE)

## Identification

Number of additional studies identified through other sources including contact with researchers: 0

Number of eligible Studies for which IPD were not sought: 0
Reasons for not seeking IPD should be reported

123 studies extracted screened by abstract

## Screening

Number of studies for which IPD were provided: 9

Number of participants for whom data were provided: 14

Number participants for whom no data were provided (give reasons:):0

Number of studies excluded: 47

Reasons: No intervention performed, intervention performed but extracranial, GCA diagnosis not confirmed

The PRISMA IPD flow diagram

© Reproduced with permission of the PRISMA IPD Group, which encourages sharing and reuse for non commercial purposes

Number of studies for which aggregate data were available : N/A

Number of participants: N/A

Number of studies for which IPD were not provided (give reasons): 0

Number of participants: N/A

Reasons for not providing IPD should be stated

**IPD (report for each main outcome)**

Number of studies included in analysis: 9

Number of participants included in analysis: 14

Number participants excluded (give reasons): 0

Participants for whom no data were provided (n= )

9 studies further analyzed for individual patient level data

55 unique studies after duplicates removed

**Aggregate data (report for each main outcome)**

Number of studies included in analysis: 0

Number of participants included in analysis: 0

Number participants excluded (give reasons): 0

Participants for whom no data were provided (n= )

## Available data

## Obtaining data

## Eligibility

## Analysed data
